# Resistant Starches and Non-Communicable Disease: A Focus on Mediterranean Diet

**DOI:** 10.3390/foods10092062

**Published:** 2021-09-01

**Authors:** Erika Cione, Alessia Fazio, Rosita Curcio, Paola Tucci, Graziantonio Lauria, Anna Rita Cappello, Vincenza Dolce

**Affiliations:** Department of Pharmacy, Health and Nutritional Sciences, University of Calabria, 87036 Rende (CS), Italy; erika.cione@unical.it (E.C.); alessia.fazio@unical.it (A.F.); rosita.curcio@unical.it (R.C.); paola.tucci@unical.it (P.T.); graziantonio.lauria@unical.it (G.L.); annarita.cappello@unical.it (A.R.C.)

**Keywords:** resistant starches, glycemic control, cancer, inflammation, microbiome

## Abstract

Resistant starch (RS) is the starch fraction that eludes digestion in the small intestine. RS is classified into five subtypes (RS1–RS5), some of which occur naturally in plant-derived foods, whereas the others may be produced by several processing conditions. The different RS subtypes are widely found in processed foods, but their physiological effects depend on their structural characteristics. In the present study, foods, nutrition and biochemistry are summarized in order to assess the type and content of RS in foods belonging to the Mediterranean Diet (MeD). Then, the benefits of RS consumption on health are discussed, focusing on their capability to enhance glycemic control. RS enters the large bowel intestine, where it is fermented by the microbiome leading to the synthesis of short-chain fatty acids as major end products, which in turn have systemic health effects besides the in situ one. It is hoped that this review will help to understand the pros of RS consumption as an ingredient of MeD food. Consequently, new future research directions could be explored for developing advanced dietary strategies to prevent non-communicable diseases, including colon cancer.

## 1. Introduction

**Figure 1:**
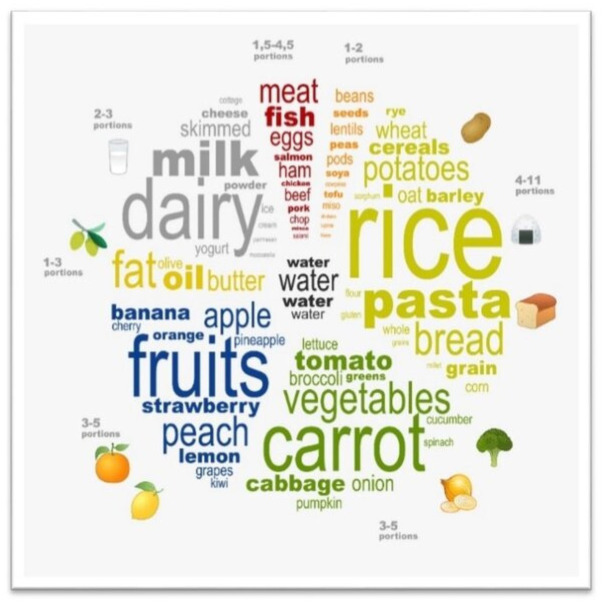
Mediterranean food pie chart.

Non-communicable diseases (NCDs) are chronic disorders, which tend to be of long duration. NCDs are due to a combination of genetic, physiological, environmental and behavioral factors, including unhealthy diets, physical inactivity, tobacco smoke or alcohol use. NCDs can be preventable by nutrition and the adoption of an active lifestyle [1,2]. Nutritional habits and physical activity represent a winning combination to counteract the rising burden of NCDs. Both unhealthy diets and a lack of physical activity are determinants to develop obesity, which in turn is linked to: (i) rise of blood pressure, (ii) increase of blood glucose and (iii) elevation in blood lipids, i.e., all conditions leading to the development of metabolic diseases. Therefore, obesity is strongly associated with chronic inflammation, which in turn leads to metabolic and cardiovascular diseases and even cancer [3]. On this basis, understanding the mechanisms leading to obesity is essential to develop preventive strategies and new treatments [4,5]. Food intake plays a critical role in the development of metabolic diseases. Recently, an investigation on European children from six different nations has highlighted that the consumption of fiber-rich foods does not meet present recommended daily intake guidelines [6]. The authors have found out a weak but significant correlation between an increase in BMI and a decrease in consumption frequency of wholegrain cereals and wholemeal products (bread, cereals, biscuits, pasta, rice). Furthermore, adopting the consumption of high-fiber foods in early childhood can delay the initiation of impaired condition [6,7]. These results are mostly driven by Italy (lower cereals consumption frequency). Angel Keys was the first to describe the Mediterranean Diet (MeD) as such and to present it to the popular thoughts as it is today. He was a biologist and physiologist who focused his studies on the dietary habits of people living in the South of Italy. Following the joint candidacy of Italy, Spain, Greece and Morocco, succeeded by Cyprus, Croatia and Portugal, the MeD was recognized by UNESCO, WHO and FAO. Such a diet varies by country and region; therefore, it has a range of definitions. It usually includes a low intake of meat and eggs, a moderate intake of dairy products and milk, and a large consumption of vegetables, fruits rich in phenols, fish, olive oil and seeds, rich in unsaturated fats [8,9]. Furthermore, legumes, whole cereals, potato, bread and rice are indicated in the Mediterranean food pie chart (Figure 1).

Taken together, those latter foods positively influence blood glycemia since their soluble fiber contents lower their glycemic index (GI), and gut bacteria ferment them. In this view, recent MeD intervention was proven to alter the gut microbiome in older people, reducing their frailty and improving health status [10]. Among the soluble fibers able to act as prebiotics, resistant starch (RS) present in foods from the Mediterranean area assumes certain importance in the MeD. This review will discuss the MeD with a particular focus on RS as a strategy to prevent NCDs, including cancer.

## 2. Type of Resistant Starch and Its Content in Mediterranean Food

According to the cause of digesting resistance, Englyst et al. classified resistant starch (RS) into four categories [11]. Later on, a new type of RS was found, which became the fifth kind of RS [12], leading to the new classification indicated in Table 1.

The formation of RS in food processing seems to be related to the amylose content, water availability and starch–lipid interaction, as recently reviewed by Ibrahim O. Mohamed [13]. RS3 refers to starch molecules that have undergone retrogradation or the realignment of starch molecules after gelatinization and chilling. Because retrograded starch molecules have a higher gelatinization temperature, they cannot fit into amylase’s substrate-binding site [13]. Although RS is not digestible, it interacts positively with the human body; in fact, it modulates the absorption of carbohydrates, determining a lowering of GI, and indirectly, a decrease in blood lipid levels. The main foods of the MeD containing RS are: (i) cereals (pasta, rice and bread), (ii) legumes (peas, beans, lentils and chickpeas) and (iii) potatoes. In particular, raw, unground grains contain more than 10% of RS, but they are not usually consumed. Different amounts of starch are present in pasta depending on its nature; for example, fresh pasta contains 49.1 ± 6.8 g/kg (dry weight ± SD) of starch, in which not more than 6% is represented by RS [14,15]. RS was also studied in other diets, such as that of India and in commonly consumed foods in the United States [16]. Legumes naturally contain RS from 4% to 5%, depending on their origin, conservation mode and type of cooking [17]. Legumes boiled and then kept for 24 h in a refrigerator can increase their levels of RS up to 6% of the total weight, permitting some fraction of amylose to recrystallize [18]. A worldwide used tubers in the MED is potatoes (white and yellow). Their processing conditions can affect health benefits. For example, cooling potatoes after cooking can substantially increase their amount of RS, tripling its content [19]. RS food content in Mediterranean foods is present in Table 2 and Table 3. RS can be determined through RS Assay Kit by Megazyme, Bray, Ireland, and recognized as the Official analysis method by the codex alimentarius methods (AOAC Method 2002.02, AACC Method 32-40.01, CODEX Type II Method) [20,21]. Although, RS obtained by food processing needs chemical-physical structure characterization [22,23,24,25,26,27,28,29,30]. To this, several techniques to determine their crystallinity, structural order, chain-length distribution and conformation, helicity, as well as double-helical structures are used [31,32,33,34,35,36,37,38]. These include: (i) scanning electron microscopy (SEM), (ii) differential scanning calorimetry (DSC), (iii) X-ray diffraction (XRD) analysis, (iv) solid-state ^13^C nuclear magnetic resonance (^13^C-NMR), (v) permethylation-GC-MS and (vi) Fourier transform infrared spectroscopy (FT-IR).

## 3. Resistant Starch in Human Nutritional Intervention Studies: GI and Impact on Inflammation and Gut Microbiome

**Figure 2:**
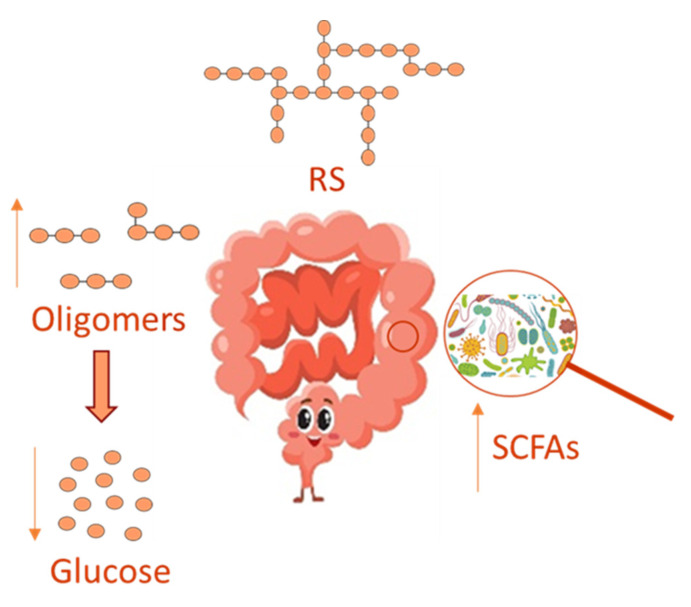
Resistant starch effect on the GI and microbiome SCFAs synthesis.

In recent years, the gut microbiota has been widely investigated, and its imbalance, due to antibiotics use [41], has been related to many disorders, including inflammation and oxidative stress, which underlie several chronic diseases, such as obesity, type 2 diabetes and chronic kidney disease [42]. Several reports have evidenced that a prebiotic supplemented diet can healthily regulate the gut microbiota, thus relieving disorders due to its imbalance. Prebiotics comprises non-digestible dietary soluble fiber, which can be used by the gut microbiota for fermentation. In this context, RS can act as a substrate for microbial fermentation in the large intestine [42] by supplying an energy source and fermentative products, such as short-chain fatty acids (SCFAs) in their anionic forms, in such a way RS is able to modulate microbial growth and could influence colonic health (Figure 2). On this basis, RS is believed to be a prebiotic and to influence the GI of foods favoring a lower intestinal absorption of glucose, even in the presence of polyphenols that in turn inhibits enterocytic starch digestion enzymes [43]

It has been reported that the extended lack of dietary fiber can lead to irreversible changes in the gut microbiota composition and elicit gut dysbiosis, even impairing gut inflammatory mediators, thereby inducing several bowel diseases [44].

### 3.1. Resistant Starch and Enhancement of Glycemic Control

When compared to food containing only readily digestible starch, the rate of digestion of RS-containing foods in the small intestine is substantially slower. As a result, consumption of such food leads to a sustained and lower level of glucose release [45]. This effect is reflected by the GI, a ranking system that organizes different food products based on the glycemic response to food consumption [45]. Researchers discovered a decrease in starch digestibility in treated food compared to untreated food after producing retrogradation in test meals [46]. They also observed a slower rise in blood glucose levels in human subjects upon consuming treated food when compared to those consuming untreated food [46,47]. Several studies have reported that potatoes generally have medium to high GI, which has often negatively impacted their consumption, but such studies have overlooked the many nutritional and health benefits of potatoes [48,49]. Interestingly, the GI varies depending on the potato variety, origin, maturity and processing methods, which can alter the starch digestibility of consumed foods. Hence, the concept of glycemic load (GL) was developed to simultaneously describe the quality (i.e., GI) and quantity of carbohydrates in a meal or diet [50,51]. Potato starch consists of 70–80% amylopectin, which is a highly branched, high molecular weight biopolymer. Amylose represents approximately 20–30% of starch, and it is a relatively long, linear, α-glucan with only a few branches. The relative proportion of amylose and amylopectin is important, given that amylose acts as a restraint to swelling and, upon cooling, it forms retrograded starch more readily [52,53,54]. Other than total starch, structure and moisture content [55], as stated above, other factors can also impact the glycemic response, including the growing conditions, maturity of the potato variety and cooking methods [56]. Potato varieties, maturity level, starch structure, methods of food processing and composition of the meal affect the GI of potatoes. Boiling, baking, microwave or oven cooking, extrusion and frying result in diverse degrees of gelatinization and starch crystallinity in potatoes. Cooling or storage after processing potatoes significantly reduces their GI because of the retrogradation of starch molecules. Although beneficial effects of resistant starch consumption have been observed, the current results of studies on the underlying molecular mechanisms in both animals and humans are not yet conclusive. Additional research efforts are necessary in order to reach a better understanding of the effects of habitual RS consumption on glycemic control.

### 3.2. Resistant Starch, Gut Microbiome and Inflammation

The mammalian gastrointestinal microbiota makes important contributions to the health of the host, including immune system development, nutrient metabolism and absorption, drug metabolism as well as protection against infection [57]. An altered microbiota (dysbiosis) has been associated with human diseases, such as diabetes, obesity, inflammatory bowel diseases, fecal occult blood and colorectal cancer [57,58,59]. By the way, diet is also considered a key modulator of the composition and function of the gut microbiota. Over the past few decades, the dietary intake of RS has been investigated. RS is a type of fermentable fiber considered a prebiotic since it can reach the large intestine, in which gut bacteria ferment it. RS fermentation leads to SCFAs production and pH reduction in the proximal large intestine. It has long been known that diet influences the microbial communities of the gastrointestinal tract. Although studies to understand how different classes of RS can affect microbiota are limited, it is clear that high-fiber diets greatly modulate the composition of mammalian microbiota [60]. Among the different classes of resistant starches, RS1–RS5 (Table 1), type 3 (RS3) is endowed with the strongest prebiotic properties [61]. Recent studies have explored the comparative physiological effects of diverse types of RS, investigating RS-induced changes in the microbiome that might be substantial in health and disease. Bacteria in the large intestine can be exposed to as much as 20 g of RS per day in humans [62]. RS may exert protective effects through broader mechanisms associated with fermentation. Acetate, propionate and butyrate are the most abundant anionic form of SCFAs generated from colonic microbial metabolism. SCFAs play a key role in the regulation of the inflammation process; contextually, they induce protective effects by stimulating or lessening inflammatory cytokines production, as well as by inhibiting or facilitating immune cells recruitment [63]. Furthermore, the fermentation of RS in the colon results in the production of gases (methane, hydrogen and carbon dioxide), small amounts of organic acid in its anionic form (lactate, succinate and formate), branched SCFAs either in anionic form such as valerate and butyrate. This latter is mainly obtained from the fermentation of RS2 than that of other RSs; notably, it has displayed interesting anti-inflammatory properties [62,64,65]. In particular, RS2 can promote a greater growth of bacteria belonging to the families of *Bifidobacteriaceae* and *Lactobacillaceae*, which are known to reduce inflammation [64]. Additionally, RS2 supplementation led to an increase in the gut level of *Faecalibacterium*; this prompted the authors to hypothesize a bacterial involvement in the anti-inflammatory effect exerted by the prebiotic fiber [65]. A future research direction to better assess gut/fecal microbial composition and serum concentration of anionic form SCFAs before and after RS2 intake could be represented by rodent models [66,67].

In any case, thanks to the healthy properties of RS, several nutraceuticals containing it are commercially available. A recent, randomized, placebo-controlled clinical trial evaluated the effects of resistant potato starch (RPS; MSPrebiotic^®^) containing 28% of fiber, administrated for 12 weeks (30 g/d), in healthy adult subjects focusing on the microbiome, reporting a reduction in the abundance of *Proteobacteria*. RPS consumers had a gut microbiome containing higher *Parasutterella* (phylum *Proteobacteria*) levels than subjects consuming placebo, andsuch increases were correlated with reductions in the blood levels of low-density lipoproteins. On this basis, it is feasible that the effect of *Parasutterella* on the host’s metabolism might depend upon several partly unknown factors, including prebiotic consumption, and they could play a critical role in cholesterol homeostasis [68,69].

### 3.3. RS, Blood Lipid Profile and Cytokines Levels

The beneficial effect of a long-term (12 months) dietary intervention with increased fiber intake, including RS on humans, was assessed [69,70]. In the study, two dietary groups were investigated, RS group subjects ingested higher amounts of food rich in RS (especially cereals and legumes) in order to consume about 15 g/day of RS, while subjects in the fiber group received general advice to ingest vegetables rich in fiber, without specific advice on the intake of RS-rich foods. At the end of the study, in the RS group, anthropometric parameters, such as body weight, body mass index (BMI) and waist circumference, were slightly more decreased, and a negative correlation was found between RS intake and adiponectin level, along with a negative correlation between RS intake and blood level of resistin, a possible pro-inflammatory mediator of insulin resistance. Furthermore, leptin and apelin levels were significantly decreased only in the RS group [71,72]. As already discussed above, RS exists in different types, having different chemical structures. In a double-blind controlled, crossover intervention study, a diet enriched with RS4, a chemically modified starch, was found to lower blood cholesterol and improve body composition measured by dual-energy, X-ray absorptiometry (DXA) [73]. The RS4 enriched diet was used for 26 weeks in the management of metabolic syndrome (MetS) using RS4 flour at 30% (*v*/*v*). Regular flour was used as a control (CF). Similar results on human lipid profiles were highlighted with RS3 (40 g/d for 21 days) in overweight and obese females [73] and RS2 (>25 g/d for 12 months) in overweight and obese subjects [73,74]. High–RS potato starch (with low protein) was used as a source of RS in patients with early type 2 diabetic nephropathy (DN) for 12 weeks [75]. In the study, the control group subjects consumed each day protein restriction diet with an everyday staple, whereas subjects of the intervention group ingested 50 g of high-RS, low-protein flour instead of an everyday staple of equal quality at lunch and dinner every day. A significant reduction in the levels of hemoglobin A1c (HbA1c) and lipid profile was found in the intervention group, along with a marked decrease of serum uric acid and urinary β2-microglobulin levels [74], thus delaying the progression of early type 2 DN. Several short-term intervention studies employing whole grain (WG) foods, including WG barley or rye derivatives rich in intact kernels, dietary fiber and RS, exhibited anti-obesogenic and anti-diabetic effects in healthy subjects [39,40,75]. More recently, a short-term, crossover, randomized study investigated the effect of rye-based bread preparation on healthy middle-aged subjects by employing white wheat flour bread (WWB) as a reference in order to evaluate possible effects on cardiometabolic risk markers, cognitive functions and mood [40]. Rye-based bread was prepared using a WG rye kernel/flour mixture (1:1 ratio) supplemented with RS2 (RB + RS2). Such a dietary treatment significantly increased insulin sensitivity, fasting concentrations of plasma butyrate, acetate and total SCFAs anionic form, which in turn have beneficial systemic effects [40]. Moreover, the dietary treatment significantly increased the fasting levels of plasma gut hormones, such as the peptide YY (PYY) and the glucagon-like peptide (GLP)-2. Furthermore, fasting levels of the inflammatory marker interleukin (IL)-1β were significantly decreased. Remarkably, the fasting concentrations of butyrate and acetate and the breath hydrogen excretion were significantly increased when preceded by the RB + RS2 intervention, suggesting that increased gut microbial fermentation of dietary fiber could mediate the observed good effects. On the other hand, blood levels of lipids, and of other inflammatory markers, including C-reactive protein, brain-derived neurotrophic factor, IL-6 and IL-18, did not significantly differ between the RB + RS2 and WWB group, as well as no significant differences in appetite sensations were observed (satiety, desire to eat or hunger), and in cognitive performance. However, insulin sensitivity was found to be positively correlated with working memory test performance. Furthermore, subjects receiving the RB + RS2 intervention felt glad more, pleased, happy, active, awake and peppy when compared to those of the WWB group. Overall, studies in humans have highlighted that the dietary intake of RS seems to not directly and significantly affect body weight and composition. In the same way, the effects of RS on reducing energy intake, increasing satiety and improving lipid profiles are controversial. On the other hand, RS supplementation can healthily affect glucose homeostasis by decreasing fasting or postprandial glucose levels and improving insulin sensitivity. Additionally, RS exerts beneficial effects on the gut microbiota, and it positively modulates gut hormones, such as GLP-1 and PYY. Nevertheless, such statements are still not definitive since further research is required, especially regarding the need for a larger sample size and longer intervention times [76]. Additionally, Chang et al. showed that the anti-inflammatory properties, as well as the inhibition of pro-inflammatory responses by intestinal macrophages, performed by butyrate, were dependent on its histone deacetylase (HDAC) inhibitory activity. Such inhibition, in turn, increased histone H3 acetylation within genetic loci required for regulatory T cells (also called Tregs) induction [77]. Moreover, it was evidenced that butyrate increased the release of the anti-inflammatory cytokine IL-10 and decreased the production of pro-inflammatory cytokines, such as TNF-α, IL-6 and nitric oxide (NO) [78]. Recent studies highlighted that a diet supplemented with buckwheat RS induced a significant decrease in TNF-α and interleukin-6 (IL-6) levels in high-fat diet (HFD)-fed mice; therefore, buckwheat RS supplementation could improve inflammatory response in plasma [79]. Instead, controversial results emerged from human nutritional clinical studies regarding the RS2 effect on inflammatory mediators. Indeed, a study performed on hemodialysis patients, who had taken RS2 for 4 weeks, evidenced a decreased IL-6 concentration [80]. In agreement, in patients with systemic inflammation associated with chronic kidney disease, a supplemented diet containing high-amylose maize RS2 elicited a significant decrease in serum levels of IL-6 and TNFα with respect to the placebo [81]. On the contrary, in women with type 2 diabetes, RS2 dietary intake did not induce any significant change in IL-6 concentration compared to the placebo [82]. RS2 significantly decreased TNF-α level and ameliorated both glycemic and lipid profiles in women with T2DM after 8 weeks of treatment [83]. Conversely, after 12 weeks of supplementation with RS, prediabetes adults displayed reduced concentrations of plasmatic TNF-α but no significant improvement in insulin resistance [84]. These controversial results were recently reported in a systematic review with meta-analysis. The authors concluded that RS2 could not reduce inflammatory mediators, but they also recognized the need for more randomized controlled trials with longer intervention in terms of duration, use of higher dose and studies in different countries [85].

## 4. Potential Mechanisms of Resistant Starch in Prevention of Colon Cancer

**Figure 3:**
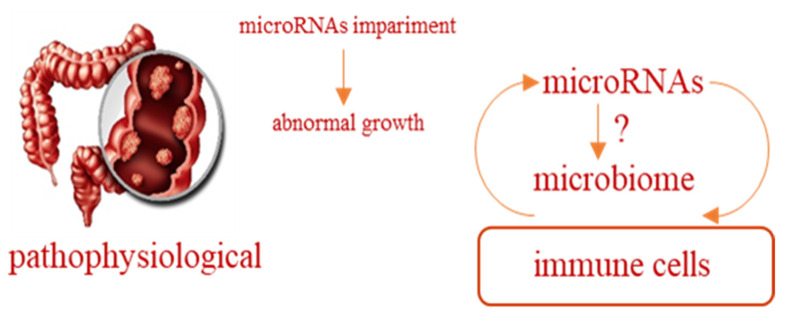
Poor resistant starch in diet as a promoting factor of colon cancer.

As discussed above, fermentation products, especially butyrate, benefit colonic health by regulating colonic enterocyte proliferation, differentiation and apoptosis. This regulation leads to a less proliferative and more differentiated phenotype and results in fewer pre-neoplastic lesions induced by colon carcinogenesis. RS diets have a significant impact on the composition of the colonic bacterial community. A recent study showed that the three major phyla predominating in the mammalian gut, Firmicutes, Bacteroidetes and Actinobacteria, showed significant changes in their relative abundance in animals fed RS [86]. Thus, the changes in the gut microbiota evoked by RS could favor microorganisms producing butyrate, which in turn serve as an energy source for the colonocytes, and they have been proposed to protect against colon cancer [87]. Colon cancer is one of the most common gastrointestinal tumors, second in women and third in men worldwide. The incidence of colorectal cancer rises with age, and it is well recognized that environmental factors, such as adiposity, poor physical activity and junk-food diet, play a major role in carcinogenesis [86,87,88]. A systematic review of the epidemiological literature has reported convincing evidence that higher intakes of red meat, processed meat, increases in body fat and alcoholic drinks increase the risk of colorectal cancer. In contrast, increased physical activity, foods containing dietary fiber and garlic, as well as calcium, may reduce the risk [88]. Although studies of RS and human colonic health are still needed, RS may protect the human colon against possibly damaging aspects of dietary red meat and have important biological effects, including colon cancer reduction [89,90]. Several potential mechanisms have been proposed by which dietary RSs are believed to alter the development or progression of colon cancer. The most common hypotheses have focused on the increase of fecal wet weight, fecal pH, defecation frequency and modification of the microbiota, with increased production of important metabolites, SCFAs, such as acetate, propionate and butyrate, which appear to have important biological effects. As already mentioned, RSs cannot be digested by amylases in the small intestine and arrive into the colon to be fermented by microbiota. One protective mechanism for RS is indeed the production of fermentation products, in particular, butyrate has raised the most interest since it may be protective against colorectal cancer [91,92]. Butyrate is a histone deacetylase inhibitor and is considered an important factor in the maintenance of the healthy function in colorectal mucosa. Normal colonocytes gain 70–80% of their energy from butyrate; therefore epithelial proliferation is supported [93]. On the other hand, on intestinal tumor cell lines, butyrate showed anti-tumorigenic effects, including reduction of cell proliferation and induction of differentiation and apoptosis [94,95]. RS led to modifications in the morphologically normal colonic mucosa, similar to those observed in cell cultures after treatment with butyrate. Recently, a rodent study highlighted that RS supplementation to a high red meat diet increased colonic butyrate levels, thus decreasing inflammation, attenuating red meat-induced DNA damage, and reducing adenocarcinoma formation in response to carcinogens [96]. In addition, animal studies identified that high butyrate levels induced by RS supplementation led to an increased expression of genes involved in DNA repair, which is expected to result in fewer mutations and reduced carcinogenesis in rapidly dividing populations of the colonic mucosa [97]. Hence, enhanced removal of damaged cells and an increased repair efficiency owing to lower proliferation could be involved in tumor prevention by RS. Butyrate’s capability to regulate gene expression results from epigenetic mechanisms, including its role as a histone deacetylase inhibitor able to modulate DNA methylation and the expression of microRNA (miRNA). Butyrate has also been shown to influence gene expression in the colon by modulating RNA splicing [98] and exerting an influence on cell proliferation and differentiation through the modulation of several signal transduction pathways, among which the most important seems to be the modulation of the Wnt signaling pathway [99]. In some colon cancer cell lines, constitutive expression of the canonical Wnt pathway, an initiating event in most colorectal cancers, is upregulated by butyrate treatment, resulting in a strong apoptotic response [100]. Gene expression is also regulated by epigenetic mechanisms, such as histone modifications or DNA methylation and the expression of miRNA. The randomized, controlled, crossover trial by Humphreys et al. [101] was the first reported human study investigating the effects of RS on miRNA expression. The study highlighted how a diet rich in red meat could increase by approximately 30% miRNA expression from the miR-17-92 cluster, an oncogenic cluster overexpressed in colorectal cancer, while expression of five miRNAs from this cluster, namely miR-17, miR-19a, miR-19b, miR-20a and miR-92a were significantly reduced in subjects fed with RS (Figure 3).

In addition to inhibiting tumor cell proliferation, butyrate might reduce colorectal cancer risk by enhancing the apoptotic response to DNA damage induced by genotoxic colorectal carcinogen [102,103]. Moreover, butyrate has been shown to affect the in vivo production and composition of mucus, which plays important protective and immunological roles, and provides the environment for colonic microbiota [104,105]. All these data may indicate a preventive role for butyrate-producing RS in the development of colorectal neoplasia. As mentioned above, RS may also exert its protective effect through broader mechanisms associated with fiber, such as by reshaping gut microbiota versus a more beneficial state, reducing bile acid metabolism, increasing fecal bulk, decreasing transit time and reducing pH levels in the colonic lumen [106,107]. Hence, altogether these effects might contribute to the prevention of and protection from colorectal cancer.

## 5. Food Claims Regarding Resistant Starch

Resistant starch is naturally found in processed and whole starchy foods belonging to the Mediterranean diet, including bread, cereals, grains, pasta, potatoes, rice and legumes. Raw foods have the highest RS content; cooked and then chilled potatoes and cereals have a higher RS content than the boiled or heated ones, as the refrigeration process favors retrogradation of starch granules to make them less digestible. Shelf life also increases RS content in some foods, such as durum wheat pasta. RS food claims were based on clinical trials [12,16,39,40,68,69,70,71,72,73,74,75].

The European Food Safety Authority (EFSA) authorized a health claim concerning the benefits of RS consumption on postprandial blood glucose concentrations [108,109]. In addition, the US Food and Drug Administration (FDA) authorized a qualified health claim for high-amylose maize RS and reduction of type 2 diabetes risk [108,109,110].

## 6. Conclusions and Future Direction

The Mediterranean diet lifestyle pattern is important to delay and fight non-communicable diseases, the assessment of resistance starches content in foods from the Mediterranean diet could improve Mediterranean diet adherence. As future research directions, the development of advanced dietary strategies highlighting the percentage of resistant starches in the nutritional label of food products would be useful. In this view, the regulatory institutions and/or governments should include the resistant starches ingredient, labelling it in the food manufacturers. This could be a strategy to make consumers safe to choose foods able to prevent non-communicable diseases, including colon cancer.

## Figures and Tables

**Table 1:** Classification of RS and example of foods rich in RS.

Classification	Description	Example
RS1	Physically inaccessible starch	Whole grains
RS2	Starch with B- or C-polymorph	Uncooked potato, high-amylose maize starch
RS3	Retrograded starch	Cooked and cooled potato starch
RS4	Chemically modified starch	Cross-linked starch in thickeners
RS5	Amylose–lipid complex	Palmitic acid-amylose complex

**Table 2:** Resistant starch in grains and legumes.

Sample	TT	TS (%)	RDS (%)	SDS (%)	RS (%)	HI	pGI	Reference
Bean flour	RAW	43.14 ± 0.14 c	82.11 ± 0.43 b	10.25 ± 0.45 i	7.64 ± 0.58 f	87.71 ± 0.44 b	87.86 ± 0.60 a	
	ANN	43.06 ± 0.33 c	77.93 ± 0.68 d	11.11 ± 0.54 h	10.96 ± 0.19 c	78.11 ± 0.59 e	82.59 ± 0.49 d	[17,18,19]
	HMT	43.65 ± 0.31 c	59.63 ± 0.65 g	25.11 ± 0.96 c	15.26 ± 0.43 a	64.67 ± 0.27 j	75.21 ± 0.11 e	
Broad bean flour	RAW	43.43 ± 0.57 c	80.26 ± 0.22 c	11.14 ± 0.44 h	8.60 ± 0.32 e	84.66 ± 0.42 c	86.18 ± 0.58 b	
	ANN	42.26 ± 0.67 d	73.75 ± 0.33 e	16.61 ± 0.55 e	9.64 ± 0.26 d	76.42 ± 0.03 f	81.66 ± 0.04 d	[17,18,19]
	HMT	42.11 ± 0.76 d	60.52 ± 0.68 g	26.19 ± 0.76 c	13.29 ± 0.43 b	68.42 ± 0.80 g	77.27 ± 0.08 e	
Chickpea flour	RAW	45.32 ± 0.29 b	85.26 ± 0.77 a	9.05 ± 0.76 j	5.69 ± 0.46 g	90.15 ± 0.39 a	89.20 ± 0.37 a	
	ANN	44.95 ± 0.87 b	77.10 ± 0.19 d	14.26 ± 0.20 f	8.64 ± 0.09 e	76.16 ± 0.85 f	81.52 ± 0.27 d	[22]
	HMT	44.99 ± 0.55 b	61.10 ± 0.37 g	27.11 ± 0.89 b	11.79 ± 0.78 b	67.21 ± 0.16 h	76.60 ± 0.49 e	
Lentil flour	RAW	47.25 ± 0.11 a	80.06 ± 0.34 c	12.68 ± 0.65 g	7.26 ± 0.61 f	82.16 ± 0.49 d	84.81 ± 0.83 c	
	ANN	47.61 ± 0.98 a	70.60 ± 0.44 f	19.26 ± 0.39 d	10.14 ± 0.65 c	75.33 ± 0.55 f	81.06 ± 0.65 d	[20]
	HMT	47.65 ± 0.54 a	59.60 ± 0.97 g	30.14 ± 0.65 a	10.26 ± 0.17 c	66.36 ± 0.47 i	76.14 ± 0.69 e	
Pea	H1	59.9 ± 1.78 c			3.7 ± 0.12			[23]
	H2				3.2 ± 0.11			
Wheat	H1	69.8 ± 1.10 d			1.9 ± 0.21			[14,15,16]
	H2				1.8 ± 0.14			
Rice	H1	81.4 ± 1.10 f			1.4 ± 0.16			[28]
	H2				1.2 ± 0.08			
Barley	H1	65.6 ± 0.76 c			2.8 ± 0.23			[39,40]
	H2				2.6 ± 0.09			
Potato	H1	85.51 ± 1.64 g			1.8 ± 0.15			[18,28]
	H2				1.7 ± 0.08			

Different letters in the same column indicate significant differences (*p* < 0.05). TT: Thermal Treatment; TS: total starch, RDS: rapidly digestible starch, SDS: slowly digestible starch, RS: resistant starch, HI: hydrolysis index, pGI: predicted glycemic index; ANN: annealing, HMT: heat moisture treatment, H_1_: conventional boiling, H_2_: pressure cooking.

**Table 3:** Starch of whole legume seeds.

Sample	TS (%)	Thermal Treatment	RS (%)	Reference
Bean	38.34 + 0.7	Boiled	4.96 + 0.9 a (12.9) *	
		Cooked	8.45 + 1.1 b (22.0) *	[17,19]
		Reheated	8.24 + 0.3 b (21.5) *	
Chickpea	41.36 + 1.0	Boiled	4.35 + 0.4 a (10.5) *	
		Cooked	5.48 + 0.2 b (13.2) *	[17,19]
		Reheated	5.58 + 0.1 b (13.5) *	
Lentil	46.72 + 2.1	Boiled	7.56 + 0.6 a (16.2) *	
		Cooked	8.60 + 0.3 bc (18.4) *	[20]
		Reheated	7.62 + 0.3 ab (16.3) *	

Results are expressed as a percentage of dry matter (mean standard deviation, *n* = 4). Different letters in the same column indicate significant differences (*p* < 0.05) between the data for each legume. * Values in parentheses are RS expressed as a percentage of TS.
